# Retrograde transcatheter aortic valve closure in an infant with failing Norwood stage I palliation: a case report

**DOI:** 10.1186/s13256-019-2141-5

**Published:** 2019-07-17

**Authors:** Hannah E. Fürniss, Rouven Kubicki, Brigitte Stiller, Katja Reineker, Matthias Siepe, Jochen Grohmann

**Affiliations:** 1 Department of Congenital Heart Disease and Pediatric Cardiology, University Heart Center Freiburg – Bad Krozingen, Medical Center – University of Freiburg, Faculty of Medicine, Mathildenstrasse 1, 79106 Freiburg, Germany; 2 Department of Cardiovascular Surgery, University Heart Center Freiburg – Bad Krozingen, Medical Center – University of Freiburg, Faculty of Medicine, Hugstetter Strasse 55, 79106 Freiburg, Germany

**Keywords:** Hypoplastic left heart syndrome, Transcatheter, Device implantation, Aortic regurgitation, Infant

## Abstract

**Background:**

Aortic valve regurgitation leading to coronary steal phenomenon can severely impair cardiac function in hypoplastic left heart syndrome, thus worsening long-term outcome.

**Case presentation:**

A German infant with borderline aortic and mitral valve, hypoplastic left ventricle, ventricular septal defect, and hypoplastic aortic arch with critical coarctation initially underwent aortic arch reconstruction and aortic valve dilation with the aim of biventricular correction later on. Unfortunately, severe cardiac dysfunction necessitated a change in strategy entailing modified stage I Norwood palliation. Increasing aortic regurgitation with coronary steal was revealed postoperatively, which required redo surgery to oversew the valve. However, pronounced aortic regurgitation recurred, causing severe cardiac decompensation with repeated resuscitation. As a bailout strategy, we performed aortic valve closure via transfemoral retrograde implantation of an Amplatzer Duct Occluder II device. This led to the patient’s rapid stabilization while circumventing highly risky renewed surgery in such a critically ill infant.

**Conclusions:**

Retrograde transcatheter aortic valve closure may be considered a feasible alternative in infants with a failing single ventricle due to aortic regurgitation, with critical device evaluation being crucial for successful device implantation in this young age group.

**Electronic supplementary material:**

The online version of this article (https://doi.org/10.1186/s13256-019-2141-5) contains supplementary material, which is available to authorized users.

## Introduction

Hypoplastic left heart syndrome (HLHS) is characterized by variably underdeveloped left-sided heart structures, mostly leading to single-ventricle physiology. Treatment options are limited to heart transplantation or a three-stage palliation concept. Though rare in HLHS, aortic regurgitation (AR) can lead to a coronary steal phenomenon, worsening morbidity and mortality significantly [1]. Although AR in HLHS has primarily been treated surgically in the past, interventional concepts have emerged in the last decade [2, 3]. In this case report, we present a novel, retrograde approach for transcatheter aortic valve (AoV) closure as a bailout strategy for AR in HLHS.

## Case presentation

A full-term male German neonate (birth weight 3110 g) was diagnosed postnatally with borderline mitral and aortic valve (z-score −2 for both) and hypoplastic left ventricle (LV), as well as hypoplastic aortic arch with critical coarctation (CoA), perimembranous ventricular septal defect (VSD; size 3–4 mm), and patent ductus arteriosus (PDA). His LV function was significantly impaired, so we did not take a primary corrective surgical approach initially. To preserve the option for later biventricular correction, we performed CoA resection and PDA closure on the 11th day of life with the aim of improving systemic blood perfusion and allowing for growth and functional improvement of the left-sided heart structures over time. Due to increasingly severe stenosis of the AoV, the patient underwent retrograde balloon valvuloplasty of the AoV at 3 weeks of age. However, although ballooning had moderately reduced the gradient, severe biventricular dysfunction developed as a result of the persistent pressure overload. In an emergency setting, a modified stage I Norwood palliation (aortic arch augmentation, Blalock-Taussig shunt, atrioseptectomy, VSD enlargement, resection of a subaortic membrane) was performed to acutely relieve the ventricles when the patient was aged 5 weeks. Later, increasingly severe AR of the native AoV became apparent, leading to recurring cardiac decompensation. This necessitated redo surgery with the AoV being oversewn 8 days after the stage I procedure.

**Fig. 1:**
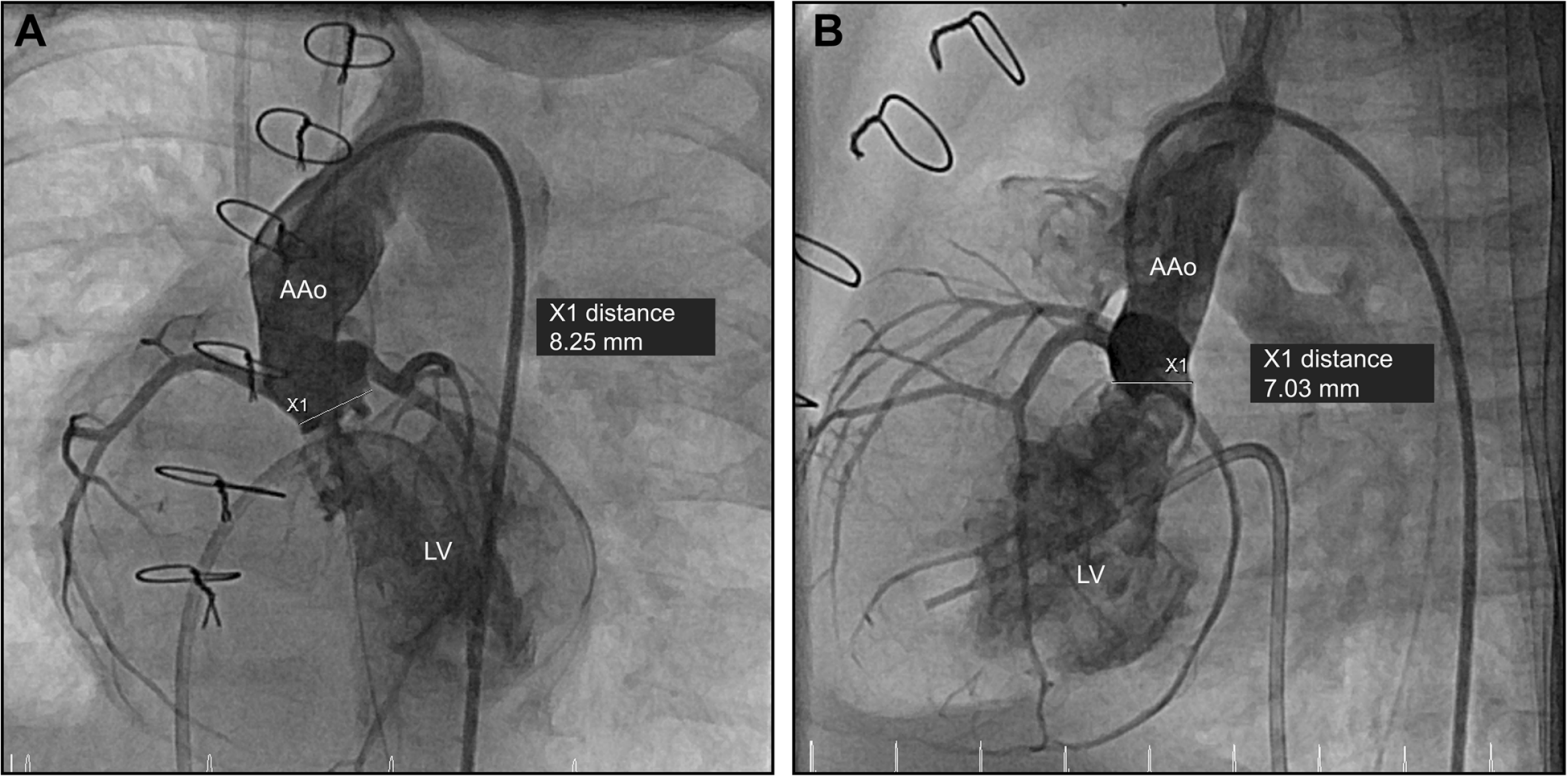
Angiograms of the ascending aorta in left anterior oblique (**a**) and lateral (**b**) views revealing severe aortic regurgitation. Aortic valve’s “ring” diameter is measured as 8.3 × 7.0 mm. *LV* Left ventricle, *AAo* Ascending aorta

**Fig. 2:**
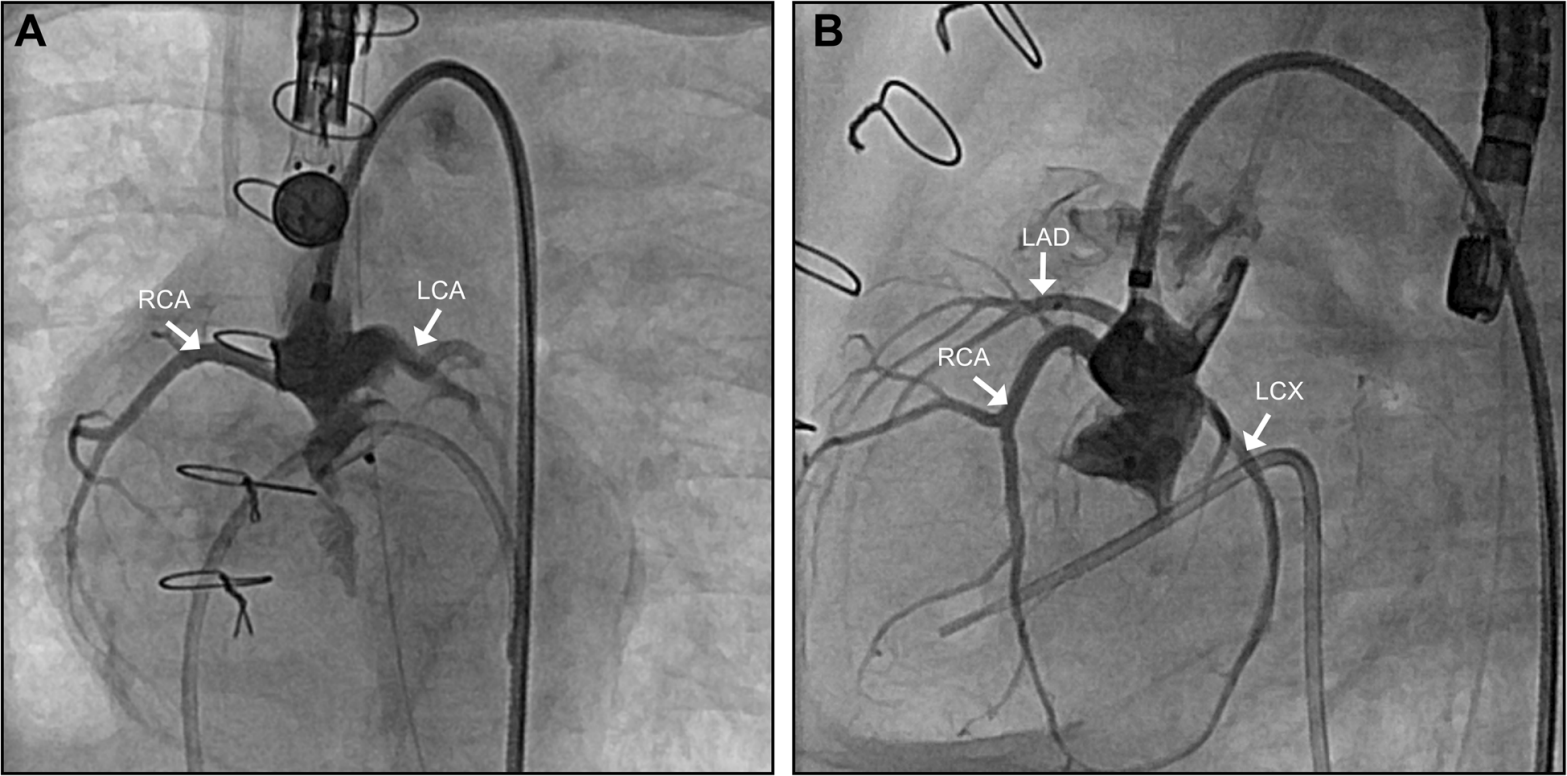
Angiograms taken while positioning the device in left anterior oblique (**a**) and lateral views (**b**) demonstrating unobstructed coronary arteries (white arrows). *RCA* Right coronary artery, *LCA* Left coronary artery, *LAD* Left anterior descending artery, *LCX* Left circumflex artery

**Fig. 3:**
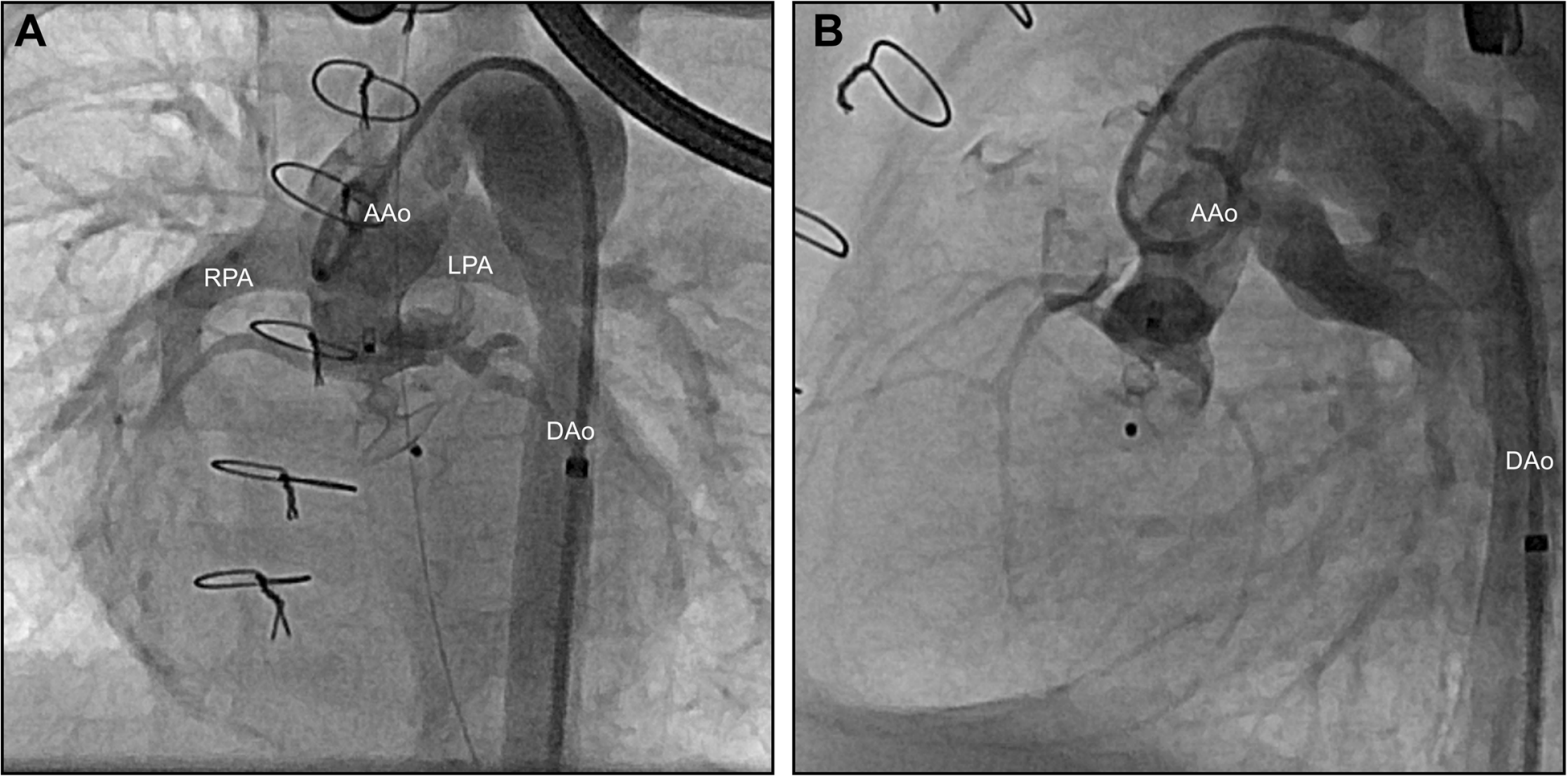
Angiograms of the ascending aorta after release of the device in left anterior oblique (**a**) and lateral views (**b**) with stable device position and minimal residual regurgitation. *AAo* Ascending aorta, *DAo* Descending aorta, *RPA* Right pulmonary artery, *LPA* Left pulmonary artery

Nine weeks later, we observed recurrent, significant AR (Fig. 1; Additional file 1: Video S1), with the patient demonstrating signs of impaired coronary perfusion in stressful situations, including repeated events of cardiopulmonary decompensation requiring resuscitation. Due to this unstable hemodynamic situation, we opted against further surgery in favor of transcatheter AoV closure. The patient’s body weight at this time was 4100 g. To accommodate the native AoV’s “ring” diameter of 8.3 × 7.0 mm (Fig. 1), we selected an Amplatzer Duct Occluder II 3–4 (ADO II; Abbott, St. Paul, MN, USA) with 9-mm discs. A 4-French Cook Flexor 45-cm-long guiding sheath (Cook Medical, Bloomington, IN, USA) was used to deliver the device to the left ventricular outflow tract (LVOT) via the right femoral artery. Positioning of the ADO II into the aortic root and LVOT was guided by transesophageal echocardiography and fluoroscopy. Repeated aortic root angiograms prior to device release safely ruled out coronary artery obstruction by the aortic disc (Fig. 2; Additional file 2: Video S2). An electrocardiogram demonstrated continued absence of atrioventricular conduction block. After the device’s release, we confirmed stable device position in the aortic root and LVOT with minimal residual AoV regurgitation (Fig. 3; Additional file 3: Video S3), preserved biventricular function, and unobstructed flow over the VSD (Additional file 4: Video S4). During the intervention, the patient was heparinized, followed by dual-antiplatelet therapy. He was extubated 5 days after the intervention without presenting any signs of respiratory distress or coronary ischemia.

At the age of 4 months, a successful Glenn operation was achieved. However, over the long term, the infant’s respiratory situation remained problematic, and he could not be permanently weaned from mechanical ventilation. Ultimately, the patient died at the age of 6 months due to pneumonia with subsequent sepsis and cardiac decompensation (Additional file 5). Signs of endocarditis or impaired coronary perfusion were not apparent clinically or echocardiographically at any time point after device implantation.

## Discussion

We describe successful percutaneous retrograde AoV closure via the femoral artery with an ADO II device in a 3-month-old infant with borderline LV and failing Norwood stage I palliation. Severe native or acquired AR leading to impaired coronary perfusion is a rare but potentially life-threatening complication in HLHS. Additional anatomical features promoting LV decompression during diastole, such as a VSD, can aggravate the steal effect [4].

Interventional AR therapy is emerging as an alternative to operative AoV closure [2, 3, 5]. Previously, successful retrograde LVOT closure with an Amplatzer Vascular Plug 4 to treat AR in a child with stage II palliation [2] was described. Moreover, another case demonstrated the feasibility and effectiveness of AoV device closure via an antegrade transvenous approach with an Amplatzer Septal Occluder using a 6-French long sheath in an infant with failing stage I palliation [3]. We opted for a retrograde transarterial approach with a duct occluder that has not yet been described in the MEDLINE database for interventional AoV closure in patients with HLHS. Retrograde access was enabled by the ADO II’s symmetrical design and its slim delivery system, facilitating device advancement through the infant’s delicate arterial vasculature. Unlike in the antegrade approach, retrograde access does not require crossing of the septum and the mitral valve or the formation of a straining loop in the LV to reach and cross the AoV with the long delivery sheath. The retrograde approach enables direct access to the AoV, thus greatly facilitating device implantation and making the technique more feasible in complex anatomies and preexisting impaired ventricular function.

## Conclusions

Retrograde transcatheter AoV closure is a viable bailout option in the context of failing single-ventricle palliation with significant AoV regurgitation. Risks of coronary artery obstruction, VSD obstruction, and conduction block, as well as vessel damage at the access site, can be limited by careful and individualized device choice, making AoV device closure an alternative to high-risk redo surgery in certain patients.

## Additional files

Additional file 1: **Video S1.** Angiogram of the ascending aorta demonstrating severe regurgitation of the native aortic valve. (MP4 1002 kb)

Additional file 2: **Video S2.** Angiogram of the ascending aorta demonstrating unobstructed coronary arteries during positioning of the device. (MP4 782 kb)

Additional file 3: **Video S3.** Angiogram of the ascending aorta after release of the device showing mild residual aortic regurgitation. (MP4 800 kb)

Additional file 4: **Video S4.** Angiogram of the left ventricle demonstrating preserved ventricular function and unobstructed outflow over the ventricular septal defect. (MP4 994 kb)

Additional file 5: Timeline. (TIF 95 kb)

## Data Availability

Not applicable.

## Abbreviations

ADO II: Amplatzer Duct Occluder II
AoV: Aortic valve
AR: Aortic regurgitation
CoA: Coarctation of the aorta
HLHS: Hypoplastic left heart syndrome
LV: Left ventricle
LVOT: Left ventricular outflow tract
PDA: Patent ductus arteriosus
VSD: Ventricular septal defect
