# Supplementary figures and images for: Retrograde transcatheter aortic valve closure in an infant with failing Norwood stage I palliation: a case report
Source: J Med Case Rep. 2019 Jul 17;13:217. doi: 10.1186/s13256-019-2141-5 (PMC6635989; doi:10.1186/s13256-019-2141-5)

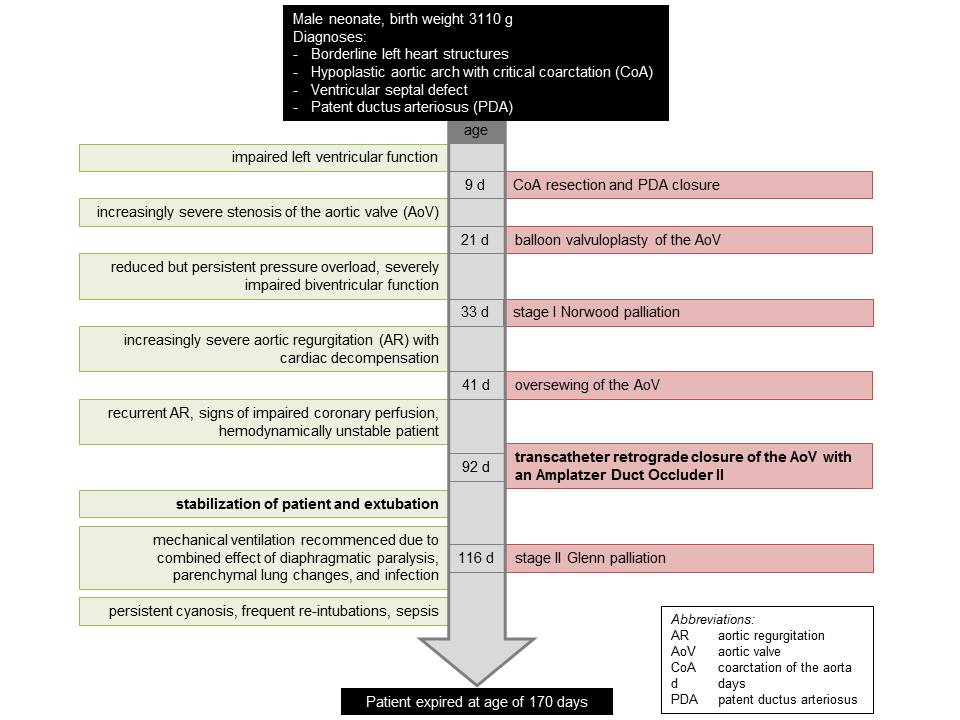

Supplement: Supplementary file 5 — Timeline. (TIF 95 kb) [file 13256_2019_2141_MOESM5_ESM.tif]
